# Supplementary material for: Analysing the Combined Effects of Radiotherapy and Chemokine Receptor 5 Antagonism: Complementary Approaches to Promote T Cell Function and Migration in Oesophageal Adenocarcinoma
Source: Biomedicines. 2024 Apr 8;12(4):819. doi: 10.3390/biomedicines12040819 (PMC11048527; doi:10.3390/biomedicines12040819)
Supplement: Supplementary file 1 [file biomedicines-12-00819-s001.zip › biomedicines-2878694-supplementary.pdf]

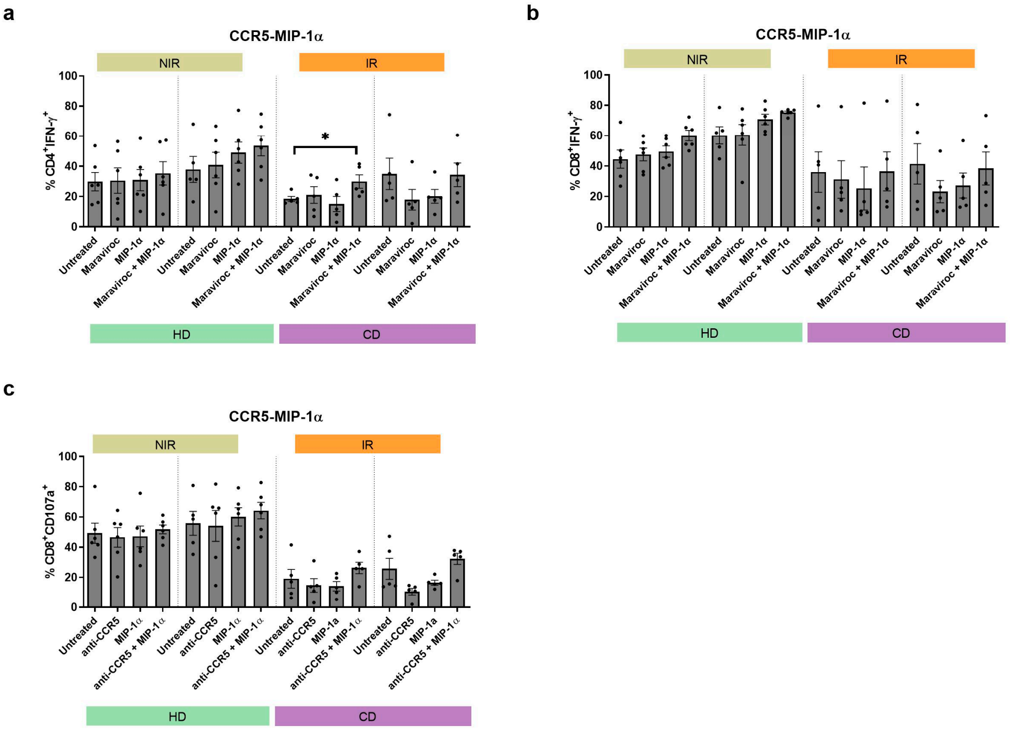

Supplementary Figure S1. The effect of the CCR5-MIP-1α axis on the production of IFN-γ by T cells and the cytotoxic potential of CD8+ T cells. PBMCs isolated from treatment-naïve OAC donors (n=5) and age-matched non-cancer donors (n=6) were activated with anti-CD3 and anti-CD28 agonists for 72 hrs and treated with Maraviroc (CCR5 antagonist) or MIP-1α or a combination of both. The PBMCs also received 2 x 1.8 Gy fractions of irradiation on day 1 and day 2, 24 hrs apart or were non-irradiated (NIR). The frequency of CD4+ and CD8+ cells producing IFN-γ (a-b) and expressing CD107a (c) was assessed by intracellular and extracellular flow cytometry. All analysis was conducted on viable T cells using a zombie dye to exclude dead cells and FMO controls were used for gating analysis. Paired parametric t test was used to compare between two groups *$p<0.05$.
